# Macro-economic determinants, maternal and infant SDG targets in Nigeria: Correlation and predictive modeling

**DOI:** 10.3389/fpubh.2022.999514

**Published:** 2022-12-12

**Authors:** Yetunde Oluranti Adegoke, Josue Mbonigaba, Gavin George

**Affiliations:** ^1^School of Accounting, Economics and Finance, University of KwaZulu-Natal, Durban, South Africa; ^2^Health Economics and HIV and AIDS Research Division (HEARDS), University of KwaZulu-Natal, Durban, South Africa

**Keywords:** health outcome, macro-economic factors, ARDL (autoregressive distributed lag), SDG, Nigeria

## Abstract

**Objectives:**

Unambiguously, Nigeria is off-track in achieving the health-related SDGs. Consequentially, this study aligns with SDG 3 which calls for “*good health and wellbeing for people by ensuring healthy lives and promoting wellbeing for all at all ages”*. This article examines the combined effect of health expenditure and other key macro-economic factors on health indices such as maternal and newborn and child mortality in Nigeria. Contrary to existing literature, we formulated a model that predicts the level of macro-economic determinants needed to achieve the SDG targets for maternal and newborn and child mortality in Nigeria by 2030.

**Methodology:**

The study used Autoregressive Distributed Lag (ARDL), which is usually used for large T models. The study period spans from 1995 to 2020.

**Results:**

We found a significant negative relationship between health outcomes and macro-economic determinants namely, household consumption, total health expenditure, and gross fixed capital while we determined a significant positive relationship between health outcomes and unemployment. Our findings are further supported by out-of-sample forecast results suggesting a reduction in unemployment to 1.84 percent and an increase in health expenditure, gross fixed capital, household consumption, control of corruption to 1,818.87 billon (naira), 94.46 billion (naira), 3.2 percent, and −4.2 percent respectively to achieve SDG health targets in Nigeria by 2030.

**Policy implication:**

The outcome of this result will give the Nigerian government and stakeholders a deeper understanding of the workings of the macro-economic factors, concerning health performance and will help position Nigeria, and other SSA countries by extension, toward reducing maternal mortality to 70 per 100,000 and newborn and child mortality to 25 per 1,000 births by 2030. The African leaders should consider passing into law the need for improvement in macro-economic factors for better health in Africa. We also recommend that the Nigerian government should steadily increase health expenditure to reach and move beyond the forecast level for improvement in maternal and infant mortality, given the present low and unimpressive funding for the health sector in the country.

## Background to the study

Health is recognized as an important aspect of human and economic development for both developing and developed countries. Therefore, the United Nations (UN) Millennium Development Goals (MDGs) from 2000 to 2015 and the Sustainable Development Goals (SDGs) from 2015 to 2030 underscore the importance of good health for economic development and socioeconomic progress. Developed countries are increasing their investment in health expenditure and reforms to improve health outcomes and accelerate progress toward achieving Universal Health Coverage (UHC) (1). In Africa, the political will of the national leaders to improve health was demonstrated through the Abuja Declaration meeting held in Nigeria in 2001, which called for governments to increase their funding for health. Despite the Abuja Declaration, the maternal mortality rate in Nigeria in 2015 was 856, and in 2020, it reduced to 512. This drop in the maternal mortality rate was among the highest in sub-Saharan Africa (SSA), with 20 percent of the world's maternal deaths occurring in Nigeria.

Given the peculiarities of the Nigerian health sector (high maternal and child mortality), we incorporated macro-economic factors into the health framework and formulated a model that predicts the level of health expenditure and macro-economic determinants that can achieve by 2030 the SDG targets for maternal and child mortality in Nigeria. A gap exists in the literature in this regard, which this study aims to address. Adegoke et al. (2) indicate that if the government could permit an enabling environment through timely interventions by controlling corruption, inflation, and other economic problems, it is possible to reduce maternal and child deaths while the lifespan of the population is enhanced, *ceteris paribus*. Therefore, it is instructive to confirm the effect of macro-economic factors and compute the level of macro-economic factors necessary to achieve the SDG targets for maternal and child mortality in Nigeria.

Indisputably, Nigeria is very far from the target of 70 per 100,000 live births stipulated in the United Nations SDGs target for maternal mortality. The SDGs also aim to reduce the mortality of children under-five to 25 deaths per 1,000 live births by 2030. The under-five mortality rate in Nigeria in 2020 was 59, which was above the target of 25. While, life expectancy at birth in Nigeria was 54.8 in 2020, in similar sub-Saharan African countries like Mauritius, Rwanda, Botswana, and Senegal, it was 75, 70, 69.8, and 68.8 respectively. Apart from the high mortality and low life expectancy in Nigeria, accessibility to healthcare is another crucial issue. According to Amedari and Ejidike (3), the lack of access to quality healthcare was linked to the wasteful use of primary care services at referral centers, the absence of adequately functioning primary health centers (PHCs), and corrupt practices in the health sector. Their study identified corruption as one of the determinants of health accessibility in Nigeria. Therefore, the health–macro-economic nexus could have a mediating impact on access problems through the control of corruption because the issue of corruption cuts across all levels, i.e., the government personnel and health personnel in the country cannot be fully exempted from corruption. For example, corrupt government personnel may not approve the establishment of adequate health centers that can benefit the citizens, and corrupt health personnel could sabotage the effort of the government in providing health facilities and materials. If the issue of corruption, which represents one of the ingredients for a functional macro-economic structure, can be resolved, then access problems in the health sector will be automatically controlled.

Given the prevailing health challenges in Nigeria, it is clear that the rates of improvement in the health outcomes in the country are far below the SDG targets, particularly SDG 3.8 on universal health coverage, and this is quite worrisome. Currently, Nigeria has a high child and maternal mortality coupled with inadequate health coverage and an unfavorable macro-economic environment marked by a high inflation rate, poor education, low investment rate, and high corrupt practices. This calls for an accelerated effort to achieve sustainable improvement in health outcomes. An analysis of the health-macro-economic nexus may be needed to determine if the core macro-economic factors could impact maternal and infant health outcomes for better performance. Most of the prior investigators considered only the effect of public health expenditure on health outcomes, and the increase in public health expenditure alone may not achieve the desired impact on maternal and infant mortality. The few studies that incorporated other determinants of health outcomes limited their selection to GDP per capita and socioeconomic factors (4, 5), and have emphasized the impact of macro-economic factors on health outcomes proxied as life expectancy at birth and under-five mortality, with an exclusion of maternal mortality. Moreover, no study has yet rendered an out-of-sample forecast for the macro-economic determinants and public health expenditure based on the United Nations Sustainable Development Goals for the year 2030 for maternal and child mortality. Drawing from the work of Adebayo et al. (6) and Xie et al. (7), the objectives of this research are:

- Examine the combined effect of health expenditure and other key macro-economic factors on health indices such as maternal and infant mortality in Nigeria.
- Compute the level of health expenditure that could achieve the SDG targets for maternal and infant mortality in Nigeria by 2030.
- Compute the level of macro-economic variables that could achieve the SDGs target for maternal and infant mortality in Nigeria by 2030.

## Motivation of the study

In Nigeria, public health expenditure has increased without a corresponding improvement in health outcomes as proxied by maternal mortality, infant mortality, and life expectancy at birth. Nigeria has been the fourth leading country in Sub-Saharan Africa in terms of maternal deaths since 1998. In Nigeria, maternal deaths from 1990 to 2017 averaged 163.1 million, which is quite large when compared to other SSA countries. In 2000, the Nigerian government spent 2.64% of the GDP on health. In 2005, the expenditure on health increased to 3.81%, while in 2018, it increased to 3.9%, which is about 340.45 billion nairas (8). The inability of the Nigerian government to achieve efficiency of production despite the increase in public health expenditure may be attributed to the failure to account for the impact and magnitude of the macro-economic factors (i.e., health expenditure and other factors). The efficiency of production in the Nigerian health sector can be described as the ability of a health system's management unit to generate the maximum health service outputs from a given set of inputs (9).

Although in 2001 the African leaders recommended investing 15 percent of the total budget in health and the same year the Macro-Economic Commission recommended an investment of 12 percent of GDP (10), the maternal and infant mortality rates in Nigeria have remained high among other countries from sub-Saharan Africa.

Despite many articles on the health-expenditure nexus in Nigeria (11–15), it was discovered that only a few studies [like (4, 5)] emphasized the impact of macro-economic factors on health outcomes proxied as life expectancy at birth and under-five mortality, with the exclusion of maternal mortality. Maternal mortality may have been excluded because the other authors believed it was unimportant in determining health outcomes, even though Nigeria had the highest maternal mortality rate in the world, trailing only South Sudan, Chad, and Sierra Leone. Moreover, the other studies on macro-economic determinants of health outcomes were specifically done for developed countries [see (16–19)]. Thus, this study contributes to the research literature in three distinct ways.

First, it captures the impact of macro-economic factors on maternal and infant mortality because the exclusion of maternal mortality in a Nigerian health outcome model may be faulty. After all, maternal mortality is a major health challenge in Nigeria. Second, this study incorporates core macro-economic factors like unemployment, household consumption, gross capital formation, total health expenditure, control of corruption, and education that can explain the intricacies of elements (20). Third, this study renders an out-of-sample forecast for the macro-economic determinants based on the SDG targets for maternal and newborn and child mortality because the ability to forecast the magnitude of the macro-economic factors will guarantee better health outcomes that will position the country to achieve its SDG targets and universal health coverage, which is uncommon in the literature.

This article is organized as follows; following the introduction is the motivation, the literature review and the theoretical framework are documented in section Literature review. Section Theoretical framework and methodology addresses the methodology and model estimation, and the discussion of results. Section Policy implications, recommendations, and conclusion contains the policy implications, recommendations, and conclusion.

## Literature review

### Conceptual review

#### Nigeria's health and macro-economic factors

Nigeria is regarded as the economic giant of Africa, but the country has not performed up to the expectations and desires of the citizens of this great country, especially in terms of health financing and health outcomes. Between 2000 and 2016, the government spent only 0.58 percent of the GDP on health, which is rather low given its high population. While public health expenditure remained at 0.58 percent of the GDP in 2018, maternal deaths in 2017 were 917, while child mortality was 100, with a life expectancy of 53 years, which is one of the lowest in sub-Saharan Africa. In 2017, the productivity growth rate was as low as 0.52 percent, with an inflation rate of 16 percent. In 2018, the GDP growth rate was 1.92 percent. Nigeria is considered a country with great potential, but the country has not been able to translate its endowed physical and human resources into better health outcomes, possibly because less attention is given to health. It has been investing a higher proportion of its income in overseas healthcare while the health facilities available in the country are outdated and inadequate and there is an incessant strike by the doctors due to poor remuneration (21, 22).

#### Structure of the Nigerian health system

The structure of a healthcare system can be described as the organization or arrangement of healthcare units. Better still, it is an organogram reflecting who, where, and when to access the health facilities. In Nigeria, the health structure is divided into three.

The federal government is responsible for the tertiary healthcare system, usually called teaching hospitals. The teaching hospitals operate based on referrals. Often, patients are expected to have consulted the lower levels of the health system before tertiary healthcare because teaching hospitals deal with large volumes of patients with severe cases. The tertiary health systems are better equipped with state-of-the-art equipment and skilled medical practitioners than the other healthcare units. The federal government also directs the activities of primary and secondary healthcare centers.

The state government directs the secondary healthcare system and it exists as general hospitals, comprehensive health centers, and specialized hospitals. At the secondary level, both public and private health practitioners are involved.

A primary health structure (PHS) or primary healthcare is the grassroots healthcare center and the first point of contact for patients in Nigeria. At this level, patients are attended to by nurses and community health workers rather than qualified doctors. In a situation where qualified doctors are not available at primary healthcare centers, people tend to self-refer themselves to the next level of healthcare structure.

### Empirical evidence

#### The relationship between public health spending and health outcomes

Ogbuagu and Olunkwa (11) studied the relationship between capital health expenditure and infant and maternal mortality ratios (MMR) by adopting the Autoregressive Distributed Lag (ARDL). The study produced a mixed result in which in the short run the relationship between health expenditure and income was positive and in the long run it was negative. Ogunjimi and Adebayo (12) examined the relationship between health expenditure, health outcomes, and economic growth in Nigeria from 1981 to 2017. This study adopted the Toda-yamamoto causality framework and it showed a unidirectional causality from health expenditure to infant mortality while there was no causality between real GDP and infant mortality. It also showed a unidirectional causal relationship between health expenditure and real GDP and life expectancy and maternal mortality, and a unidirectional causal relationship between real GDP and health expenditure. The study also used the ARDL to investigate if a long-term relationship exists among the macro-economic variables used, and the result was affirmative.

Eboh et al. (14) investigated the impact of public health expenditure on the infant mortality rate in Nigeria. The study made use of an ex-post facto research design and time series data spanning a period of 24 years (1994–2017), sourced from the Central Bank of Nigeria statistical bulletin 2016 and the World Bank. Descriptive statistics were used to analyze the data while the ordinary least squares technique was used to estimate the model. The study's findings revealed that the Nigerian government's health recurrent and capital expenditure had a significant negative effect on infant mortality rates over the 24-year period under consideration. Also, health recurrent expenditure had a more significant negative effect on the infant mortality rate than health capital expenditure in this study. Aronu and Bilesanmi (23) used statistical tools such as the unit root test, Granger causality tests, and least square regression analysis to examine the impact of the federal government's spending on health, agriculture, transportation, and communication on infant and maternal mortality rates. The study revealed that the federal government's expenditure on health had a significant negative impact on infant and maternal mortality in Nigeria, which implies that as the federal government increased its expenditure on health, the rate of infant and maternal mortality decreased. Edeme et al. (15) investigated the effect of public health expenditure on health outcomes in Nigeria, as captured by life expectancy at birth and infant mortality rates, utilizing the ordinary least squares technique. The study found that public health expenditure and health outcomes have a long-term equilibrium relationship. It also revealed that a rise in public health expenditure improves life expectancy and reduces infant mortality rates. In addition, the urban population and HIV prevalence rate significantly affected health outcomes, while per capita income exhibited no effect on health outcomes. The findings, therefore, suggest that public health expenditure remains a necessary component in improving health outcomes in Nigeria. Boachie and Ramu (24) examined public health expenditure and health status in Ghana. In their study, they examined the impact of public health spending on health status in Ghana for the period 1990–2002, employing standard OLS and Newey-White estimation. After controlling for real per capita income, literacy level, and female participation in the labor market, the study found that the declining or falling infant mortality rate in Ghana was influenced by public health spending, among other factors. Thus, they concluded that public healthcare expenditure is associated with an improvement in health status through a reduction in infant mortality.

#### Macro-economic determinants of health outcomes

Agbatogun and Opeloyeru (5) investigated the impact of macro-economic factors on under-five mortality in Nigeria from 1980 to 2017 using ARDL and found government health expenditure as the only significant determinant of under-five mortality, and factors such as immunization rates, GDP per capita, literacy rates, and health workers did not affect under-five mortality. Zhou et al. (19) investigated the role of macro-economic indicators on healthcare costs in 21 emerging economies of the world using a dataset that spanned from 2000 to 2018. The data were analyzed using the generalized method of moments (GMM) and the result revealed tax revenue, labor force participation, and GDP per capita as significant determinants of healthcare cost. The healthcare cost was represented by public healthcare expenditure. The study also revealed a non-linear relationship between public health expenditure and economic growth.

Romeo (25) investigated the macro-economic determinants of health crises in 25 SSA countries and employed a logit model to analyze the data spanning from 1995 to 2012. The result revealed international migration flows, the ratio of short-term debt to currency reserves, and the organization of the healthcare system as the determinants of health crises (expressed as differences in life expectancy at birth) in SSA. Naik et al. (26) reviewed the macro-economic determinants of health and health inequalities. Based on the existing literature on determinants of health, a conceptual framework was formed and the following variables were identified as the possible determinants of health: healthcare expenditure, housing, and environmental factors such as pollution and climate change.

Thompson et al. (27) studied the effect of structural adjustment programs on child and maternal health by employing observational and quasi-experimental articles published. They found that structural adjustment programs had a detrimental impact on child and maternal health outcomes because the programs impeded access to quality and affordable healthcare and also had a negative impact on the social determinants of health, such as income and availability of quality and affordable healthcare. Ogunleye (28) investigated the relationship between health and growth and also examined socioeconomic determinants of health outcomes in SSA from 1980–2003. The study incorporated alcohol consumption, urbanization, carbon emissions, food availability, and education as the socio-economic and environmental variables that can determine life expectancy at birth and child health outcomes among the selected SSA countries. The study identified alcohol consumption, urbanization, and carbon emissions as significant determinants of health outcomes using the GMM estimation technique.

By reviewing the extant literature in the area of health outcome determinants (17), investigated the social determinants of health outcomes in the world's poorest countries. The study revealed the need to be country-specific in deciding the determinants of health outcomes in poor countries so that appropriate policies that are peculiar to country-specific circumstances can be explored. Fayissa and Gutana (29) investigated the determinants of health status in SSA. The three core determinants were economic (represented by the ratio of health expenditure to GDP and the per capita food availability index), social (represented by the illiteracy rate and alcohol consumption), and environmental (determined by urbanization rate and carbon-dioxide emission per capita index). One-way and two-way panel data analyses were carried out, and the result of the two-way panel analysis revealed that a decrease in the illiteracy rate and an increase in the food availability index positively influenced life expectancy at birth.

Subramanian et al. (16) discussed the relative importance of wealth, degree of quality, and public health expenditure on health in both developed and developing nations. The study also established a reciprocal relationship between poverty and poor health. The result of the discussion ought to have served as a policy recommendation to the government, but the study was carried out without any empirical analysis to support the claims discussed in the study. Sede and Ohemeng (30) investigated the socioeconomic determinants of life expectancy in Nigeria from 1980 to 2011 using VAR and VECM frameworks. The result revealed per capita income, education, and government expenditure on health as insignificant in determining life expectancy at birth, while unemployment and nominal exchange rates were significant determinants of life expectancy.

### Conceptual framework

**Figure 1:**
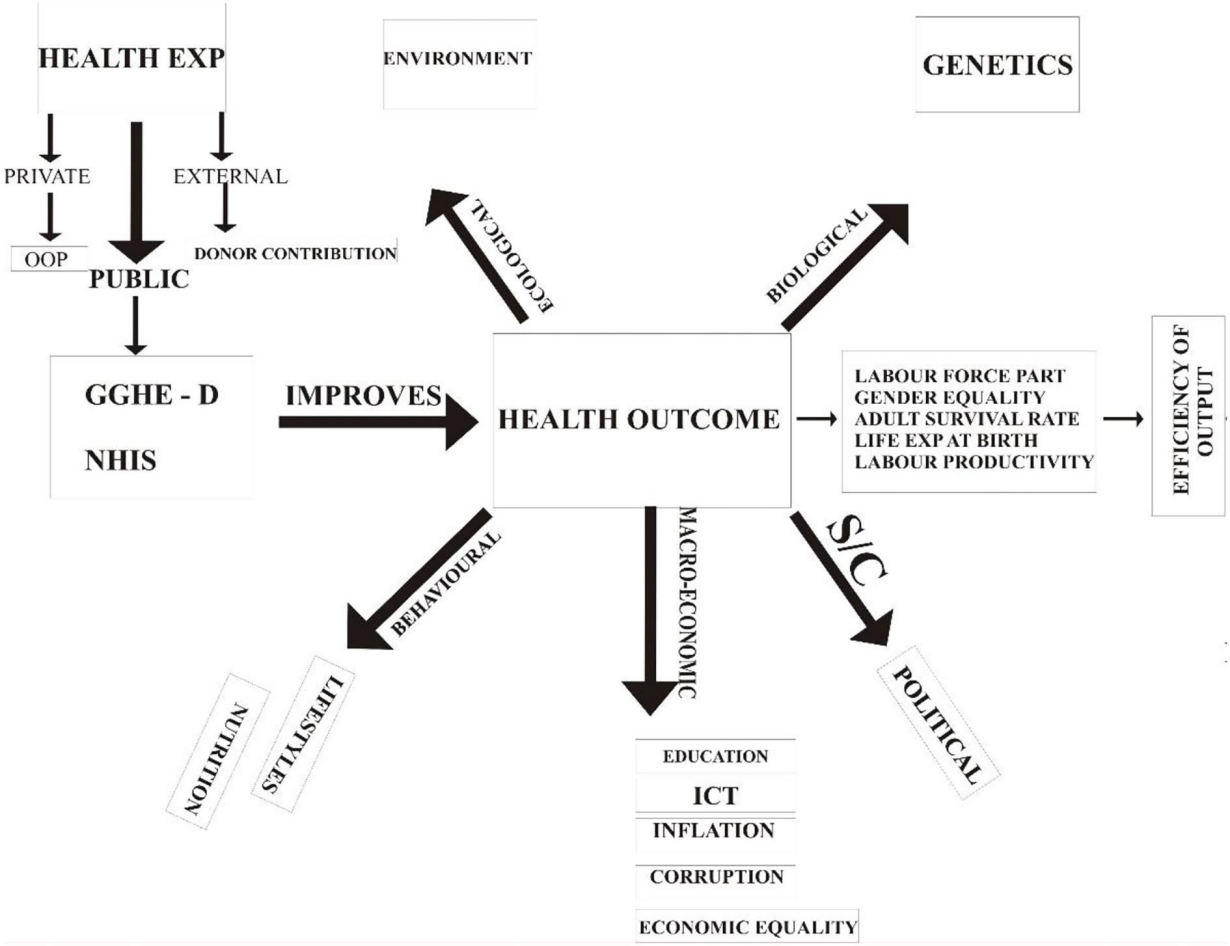
A conceptual framework connecting the effects of health expenditure and other determinants of health outcomes. Source: Authors' computation.

Figure 1 presents a brief overview of the entire study. This section explains the interconnectivity among the objectives of the study. First, the framework identifies three sources of health financing, namely, private, public, and external. Private health expenditure comprises of out of pocket (OOP) spending and private health insurance schemes; the private health insurance schemes were rated as the highest source of health expenditure in SSA, while in OECD countries public health expenditure (PHE) was the highest. The donor's contribution (DC) is the external source. PHE consists of domestic general government health expenditure and the government's contributions to the national health insurance scheme (NHIS). It is expected that total health expenditure will improve health outcomes, such as child and maternal mortality. According to the literature, health outcomes can be influenced by numerous factors. Among them are behavioral (lifestyles and nutrition), biological (genetics), ecological (environment), socio-cultural (political), and macro-economic (macro-economic) factors, which are the major focus of this study.

In this study, we considered the effects of total health expenditure, gross capital formation, control of corruption, unemployment, and household consumption on health outcomes represented by maternal and child health. It is strongly believed that these factors will positively affect health outcomes. An improvement in health outcomes will increase female labor force participation because women who might have died due to maternal complications will be absorbed into the general labor force, thereby increasing female labor force participation and labor productivity. The survival of a child will increase labor force participation and labor productivity in Nigeria because a reduction in child mortality significantly relate to higher life expectancy while life expectancy is the most remarkable determinant of modern economic development. A reduction in maternal deaths will also enhance gender equality because the Nigerian economy will have more women to compete with men, especially in male-dominated professions. A reduction in both maternal and child mortality will increase the adult survival rate and life expectancy at birth. An improvement in health outcomes will enhance the efficiency of output.

### Theoretical review

#### Materialist theory of health

The materialist theory proposes that inadequacy in an individual's income level leads to a lack of resources to cope with the stresses of life and thus results in ill health. Goldberg et al. (31); Frohlich et al. (32); Macintyre (33); Wilkinson (34), postulated that social comparison, humiliation, and shame are important mechanisms by which those who have lower social status as determined by income, education, and the nature of employment (low-status employment) have poorer health outcomes than their rich counterparts. From the perspective of the materialist theory, it can be inferred that an individual's income level will be inadequate given socioeconomic factors such as poverty, income inequality, unemployment, underemployment, lack of education, civil unrest, political instability, and poor wage structure. These factors will greatly affect the health outcome of such an individual.

#### Neo-materialist theory of health

As a subset of materialist theory, the neo-materialist approach is concerned with how nations, regions, and cities differ in the distribution of economic and other resources among the population (35). The distribution of resources can vary widely from country to country (for example, gross national income). The neo-materialist view, therefore, directs attention to both the effects of living conditions (social determinants of health) on individuals' health and how society decides to distribute resources among the citizens which indirectly affects the health outcomes of people.

#### Life course perspective

Lu and Halfon (36) provide a lens through which maternal and infant health mortality can be analyzed (36). The Life Course Perspective (LCP) theory was proposed against the backdrop of maternal challenges that result in birth outcome discrepancies (36). It borrows conceptually from early programming (37) and cumulative pathways to demonstrate how the environment influences growth (38). Both the early programming and cumulative route processes were derived from large British birth cohort research done during the 1930s (36). The early programming mechanism arose from the Barker hypothesis, which states that human development is especially sensitive to prenatal and early childhood experiences (39). It argues that these early experiences have long-term health effects, such as the development of diabetes and cardiovascular disease (37). In contrast, the cumulative path mechanism investigates the effects of accumulation and adaptation to persistent social and physical stressors, sometimes referred to in the literature as “wear and tear,” on human development and health (36, 38, 40). These stresses may include homelessness, prejudice, infectious diseases, and health-related activities [e.g., smoking; (36)]. The LCP integrates both theories by proposing that women who experience chronic stress (e.g., recurrent experiences of racism and discrimination, displacement, and intergenerational poverty) are at increased risk of adverse health outcomes, particularly when these experiences occur during critical developmental periods such as prenatal development, adolescence, and pregnancy (36, 40).

#### Historical trauma theory

The Historical Trauma (HT) theory provides an alternative lens for comprehending socioeconomic determinants by identifying the effect of traumatic experiences on the health outcomes of marginalized groups as a result of targeted oppression. It emerged from the study of Jewish Holocaust survivors as researchers investigated the phenomenon that survivors' offspring were more likely than the general population to suffer from mental health conditions such as post-traumatic stress disorder, despite not having directly experienced the Holocaust (41). This queried the ability of generations to biologically, socially, and culturally “inherit” mental and physical health disorders (42). Recent research has applied HT to Mexican Americans (43).

## Theoretical framework and methodology

### Theoretical framework

To examine the health outcomes as measured by infant and maternal mortality in Nigeria, this study follows the work of Mosley and Chen (44) who came up with a model that sees socioeconomic determinants acting through biological mechanisms to influence mortality. Based on this approach, the underlying socioeconomic (e.g., social, economic, biological, and environmental) status manifests itself in proximate determinants (maternal fertility, environmental contamination, disease control, etc.). The values of these variables influence the risk of disease, which is linked to the probability of death. Based on this framework, the factors that affect the death of mothers and children can be written as:


(1) $$\begin{array}{l} maternal=f\left(phe,cons,gfc,unemp,cor\right) \end{array}$$

(2) $$\begin{array}{l} child=f\left(phe,cons,gfc,unemp,cor\right) \end{array}$$

### Methodology

#### Model specification

We can specify the econometric model for the relationship between health outcomes (child and maternal mortality) and public health expenditure and other control variables in Nigeria as follows:


(3) $$\begin{array}{l} maternal_{t}=\beta_{0}+\beta_{1}phe_{t}+\beta_{2}cons_{t}+\beta_{3}gfc_{t}+\beta_{4}unemp_{t}\\ \;+\beta_{5}cor_{t}+\varepsilon_{t} \end{array}$$

(4) $$\begin{array}{l} child_{t}=\beta_{0}+\beta_{1}phe_{t}+\beta_{2}cons_{t}+\beta_{3}gfc_{t}+\beta_{4}unemp_{t}\\ \;+\beta_{5}cor_{t}+\varepsilon_{t} \end{array}$$

Where the notations include child mortality (*child*), maternal mortality (*maternal*), household consumption (*cons*_*t*_), gross fixed capital formation (*gfc*_*t*_), unemployment (*unemp*_*t*_), and control of corruption (*cor*_*t*_), and ε_*t*_ is the error term.

#### Estimation technique

Given the characteristics of the macro-economic variables in Nigeria, a more dynamic method of analysis that allows for different orders of integration is needed. Fortunately, Pesaran et al. (45) have developed a new ARDL model which has more advantages over other approaches (46). Significantly, the ARDL approach can be applied irrespective of whether the regressors are I(1) or I(0), or a combination of both. Also, in using the ARDL approach, dummy variables can be included in the analysis, which is not permitted in the Johansens method (47). Following the work of Kirikkaleli et al. (48) and Xie et al. (7); the ARDL models are specified to achieve the objectives of the empirical study. We specified the relationship in the ARDL (p,q) model which forms the basis for the Bounds approach to co-integration as follows:


(5) $$\begin{array}{l} △{maternal}_{t}=\theta_{1}v_{t-1}+\overset{p}{\underset{j=1}{\sum }}\lambda_{1j}△{maternal}_{t-j}+\overset{q}{\underset{j=0}{\sum }}\gamma_{1j}△{phe}_{t-j}\\ +\overset{q}{\underset{j=0}{\sum }}\delta_{1j}△{cons}_{t-j}+\overset{q}{\underset{j=0}{\sum }}\varphi_{1j}△{gfc}_{t-j}+\overset{q}{\underset{j=0}{\sum }}\omega_{1j}△{unemp}_{t-j}\\ \;+\overset{q}{\underset{j=0}{\sum }}\vartheta_{1j}△{cor}_{t-j}+\upsilon_{1t};\;t=1,\;2,\cdots \;,T \end{array}$$

(6) $$\begin{array}{l} △{child}_{t}=\theta_{2}v_{t-1}+\overset{p}{\underset{j=1}{\sum }}\lambda_{2j}△{child}_{t-j}+\overset{q}{\underset{j=0}{\sum }}\gamma_{2j}△{phe}_{t-j}\\ \;+\overset{q}{\underset{j=0}{\sum }}\delta_{2j}△{cons}_{t-j}+\overset{q}{\underset{j=0}{\sum }}\varphi_{2j}△{gfc}_{t-j}+\overset{q}{\underset{j=0}{\sum }}\omega_{2j}△{unemp}_{t-j}\\ +\overset{q}{\underset{j=0}{\sum }}\vartheta_{2j}△{cor}_{t-j}+\upsilon_{2t};\;t=1,\;2,\cdots \;,T \end{array}$$

We conducted a forecasting analysis based on the time series econometric models in Eqs. 3.5 and 3.6. The prediction was explored by forecasting values of health expenditures that are necessary to be achieved for the realization of maternal and infant mortality targets by 2030 for each of the dependent variables. In the end, we are interested in the future values of the *outcomes*_*t*_ series, that is, the forecast values of maternal and child maternity for the period *t*_2030_based on the future values of the regressors as specified in Eq. 3.7.


(7) $$\begin{array}{l} outcomes_{t_{2030}}^{*f}={\hat{\delta }}_{0}+{\hat{\delta }}_{1}phe_{t_{2030}}^{f}+\hat{\beta }macro_{t_{2030}}^{f} \end{array}$$

where $outcomes_{t_{2030}}^{*}f$ are predetermined values of maternal and child maternity based on UN standards, ${\hat{\delta }}_{0}$, ${\hat{\delta }}_{1}$, and $\hat{\beta }$ are the estimated long-run parameters, $phe_{t_{2030}}^{f}$ and $macro_{t_{2030}}^{f}$ are the predicted values of public health expenditure and other macro-economic variables for the year 2030 which will help to achieve the SDG targets of reducing infant mortality per 1,000 live births to < 25 and maternal mortality of 70 per 100,000 live births. With these forecast values as the outcomes, the required public health expenditure value that would be required to achieve Agenda 2030 can be obtained by solving the underlying estimated model for the independent variable (health expenditure) based on the known future value of the dependent variable.

#### Data sources and selection

The data was sourced from the World Development Indicators for the period 1980 to 2020. The data was selected based on the peculiar features of the Nigerian economy. The maternal and infant mortality data were selected given the huge mortality rate recorded in the country from 1980 to date. Data on unemployment, control of corruption, public health expenditure, and other macro-economic determinants were selected based on their stringent peculiarities in the country.

#### A priori expectations

We expect a negative relationship between health expenditure and health outcomes such that higher health expenditure may be expected to improve health outcomes by reducing maternal and child mortality [see (5, 49–51)]. Also, a negative relationship is expected between health outcomes and public health expenditure per capita, household consumption, and gross fixed capital. On the contrary, a positive relationship is anticipated between health outcomes, such as maternal and child mortality (as well as modern educational tools and other variables such as unemployment).

#### Discussion of the results

Despite various attempts by African leaders to increase health expenditures for better health outcomes, the health outcomes in Nigeria are perversely poor and health expenditures are inadequate. The severity of the Nigerians' health challenges calls for urgent attention that could resolve the prevailing health emergency in the country. In an attempt to proffer solutions to health challenges in the Nigerian health sector, we established a link between health and macro-economic factors. The result of the analysis provided answers to the research hypothesis that the combined effect of health expenditures and macro-economic factors will not impact health outcomes in Nigeria. On the whole, all variables of interest are significant, as shown by the estimated correlation coefficients. The strength of the relationship, in most cases, is quite strong and has the expected signs with no indication of multicollinearity among the regressors.

Table 1 contains the ARDL results on the effect of macro-economic determinants on health outcomes proxied by maternal mortality and child mortality. We found a significant negative relationship between health outcomes and household consumption both in the short run and long run. The results show that a percentage change in household consumption significantly reduces maternal and child mortality by −0.1516 and −0.1380 percent, respectively. We also identified a significant negative relationship between total health expenditure and health outcomes. An increase in health expenditure caused reductions in maternal and infant deaths. A percentage change in health expenditure significantly reduced maternal and child mortality by −0.2908 and −0.3446 percent, respectively. We also discovered a significant negative relationship between gross fixed capital and health outcomes, which means an increase in domestic investment in Nigeria will lead to reductions in maternal and infant deaths in the country. A percentage change in gross fixed capital significantly reduces maternal and child mortality by −0.1211 and −0.0123 percent respectively. The relationship between control of corruption and health outcomes is also negative but not significant in both the short run and long run. The forecasting results in Table 2 revealed the need to increase health expenditure in the country to 987.21, 1,260.57, and 1,818.87 billion naira by 2025, 2027, and 2030, respectively. In 2019, the health expenditure in the country was 474.24 billion naira, which suggests a drastic increase in health expenditure is needed in the country if the country aims to meet the SDGs on maternal and infant health by 2030. The consumption level must increase to 3.2 percent from its present level in 2019. Also, the control of corruption must increase to −4.2 percent so that the country can achieve Agenda 2030. The unemployment rate in the country was 8.5 percent in 2019. Our forecasting results suggest a reduction in the unemployment rate of 1.84 percent by 2030, and domestic investment must increase to 94.46 billion naira to enable the country to meet its SDG health targets. In 2019, the domestic investment in the country was 24.62 billion naira, which is very far from the projected level.

**Table 1:** The effect of macro-economic determinants on health outcomes.

**ARDL**	**Maternal mortality**	**Child mortality**
ECT(-1)	−0.1215*** (0.0112)	−0.1805*** (0.0139)
ΔHealth	−0.0353*** (0.0062)	−0.0622*** (0.0105)
ΔGFC	−0.0147*** (0.0052)	−0.0022 (0.0044)
ΔUnemployment	0.0017*** (0.0005)	0.0025*** (0.0005)
ΔConsumption	−0.0184** (0.0083)	−0.0249** (0.0078)
ΔCont. corruption	−0.0133 (0.0088)	−0.0128 (0.0089)
Health	−0.2908*** (0.0646)	−0.3446*** (0.0286)
GFC	−0.1211*** (0.0358)	−0.0123*** (0.0249)
Unemployment	0.0143** (0.0060)	0.0139*** (0.0033)
Consumption	−0.1516** (0.0601)	−0.1380** (0.0515)
Cont. corruption	−0.1097 (0.0705)	−0.0713 (0.0516)
F (Bounds test)	16.850***	24.211***
Serial correlation	1.6978 [0.1444]	0.9504 [0.5097]
Heteroscedasticity	1.1081 [0.4070]	1.6571 [0.1639]

This table presents the results of ARDL for the impacts of macro-economic factors on health outcomes in Nigeria. The health outcomes are maternal mortality and infant mortality. “Δ” represents short-run estimate while the absence represents long run. The standard errors associated with the coefficients are in round brackets while the *p-*values of the diagnostic tests are in square brackets. ^***^, ^**^, and ^*^ represent 1, 5, and 10% statistical significance respectively.

**Table 2:** The United Nations' 2030 SDG health targets and forecasts of public health expenditures and non-health factors in SSA countries.

**Forecasts**	**Maternal**	**Child**	**Health expenditure**	**GFC**	**Unemployment**	**Cons**.	**Corrupt**.
2025	139.4	36.9	987.21	51.27	3.70	1.74	−2.28
2027	105.5	29.2	1,260.57	65.46	2.80	2.23	−2.91
2030	69.5	20.6	1,818.87	94.46	1.84	3.21	−4.20

The SDG targets for maternal and child under-five mortality are 70 per 100,000 and 25 per 1,000 live births, respectively. The health and non-health factors are health expenditure, gross fixed capital formation, unemployment, consumption, and control of corruption.

#### Comparison with previous empirical studies and discussion of the findings

Table 1 contains the ARDL results on the effect of the macro-economic determinants on health outcomes proxied by maternal mortality and child mortality. We found a significant negative relationship between health outcomes and household consumption. Household consumption is a variable that represents the citizens' standard of living (poverty level), which means an increase in the standard of living will cause a reduction in maternal and infant deaths. This result aligns with *a priori* expectations but is inconsistent with extant literature. For instance, Kilanko (52) found an insignificant positive relationship between household consumption and health outcomes in 14 West African countries. We also found a significant negative relationship between total health expenditure and health outcomes with an increase in health expenditure reducing maternal and infant deaths, which is consistent with the existing literature [see (51, 52)].

When we get a significant negative relationship between gross fixed capital and health outcomes, it means an increase in domestic investment in Nigeria will lead to reductions in maternal and infant deaths in the country. When more money is expended on capital equipment that can enhance the health infrastructure, then the number of pregnancy-related deaths and infant deaths will be reduced. This result conforms to *a priori* expectations. The relationship between control of corruption and health outcomes is also negative [as expected, also in line with Hsiao et al. (53)] but insignificant, which means an increase in control of corruption could signal a reduction in maternal and infant deaths. However, it is insignificant, probably because of the high level of corruption existing in the Nigerian health system or the type of measurement of corruption (control of corruption) adopted in this study.

The results also showed a significant positive relationship between health and unemployment. Reductions in unemployment lead to reductions in maternal and infant mortality and vice-versa. Our findings align with *a priori* expectations and previous studies (30) on the Nigerian economy.

## Policy implications, recommendations, and conclusion

### Policy implication

- The government, enforcement agencies, and relevant stakeholders in Nigeria should establish another agency apart from the EFCC and ICPC specifically for the control of corruption in the health industry, e.g., the **National Agency for Control of Corruption in Health (NACCH)**.
- African leaders should come together to embrace the need for better macro-economic factors for health improvement just as they did in 2001 to recommend an increase in public health expenditure for better health outcomes. Despite the recommendations for an increase in health financing, the health outcomes are poor because many of the countries in Africa did not increase the portion of their income or budget allotted to health as they believed doing so would affect other sectors in their country. Our recommendation is all-inclusive because the benefits of improvement in macro-economic factors will not be limited to the health sector alone. In the process of improving the health system through better macro-economic factors, the whole economy will be improved. For example, an **improvement in macro-economic factors for better health in Africa** could be passed into law.
- Unnecessary generalizations must be avoided when dealing with health issues because each country has its own peculiar health problems that may require specific intervention. For example, the health expenditure needed to reduce maternal mortality in South Africa may not be able to reduce maternal mortality in Nigeria.

### Recommendations

Based on our analysis, we recommend:

- The government should consider macro-economic factors as key determinants of health outcomes in Nigeria because prior studies did not account for the role of core macro-economic determinants in explaining maternal and under-five health outcomes.
- We also recommend that the Nigerian government should steadily increase health expenditure to reach and move beyond the forecast level of 1.818 billion naira, specifically, if Nigeria wants to achieve the SDG targets related to maternal and infant mortality, given the present low and unimpressive funding toward the health sector in the country. While, in general, an increase in health expenditure will indirectly increase life expectancy at birth in the country, which is regarded as one of the lowest in the world; a reduction in maternal and child mortality will result in an improvement in life expectancy at birth.
- The country's unemployment rate was 8.5 percent in 2019. Our forecasting results indicate that a 1.84 percent reduction in the unemployment rate could be a reality in the country by 2030.
- Domestic investment must increase to 94.46 billion naira to propel the country toward actualizing Agenda 2030. In 2019, the domestic investment in the country was 24.62 billion naira, which is very far from the projected level.
- Household consumption represents the poverty level of the citizens. Therefore, the government must put in place strategies or reforms that can increase the household consumption level to 3.21 to achieve SDG targets for maternal and under-five child health outcomes.

## Conclusion

This study confirms total health expenditure, unemployment, household consumption, and gross fixed capital as significant macro-economic determinants of maternal and infant health outcomes in Nigeria. The results of the forecasts suggest the need for improvement of macro-economic determinants so that the country is positioned to achieve Agenda 2030. Health expenditure has been identified as a necessary prerequisite for the improvement of health outcomes (54). Health expenditure, therefore, is an all-encompassing factor that covers all the expenses incurred on health issues, prevention, pharmaceutical costs, diagnostic costs, and health-related programs (including training, research, awareness, and development), but health financing is a critical component of the health system that provides the resources needed to cover health expenditures.

In this study, our emphasis was on the total health expenditure, a combination of private, public, and donor contributions. A disaggregation of the sources of health expenditure could have enabled us to document the effect of health expenditures and macro-economic factors on health outcomes based on different sources of health expenditures.

In addition, to contribute more to the literature, we could have incorporated different measures of corruption. In this study, the focus was limited to corruption control. Different measures of corruption could have reflected the effect of corruption on health outcomes from different perspectives. For future work and subject to data availability, control variables such as GDP per capita, public health expenditure, private health expenditure, and different measures of corruption, may be considered which could change the signs, the statistical significance of the coefficients, and out-of-sample projections.

## Data availability statement

Publicly available datasets were analyzed in this study. This data can be found here: World Development Indicators, Penn World Table.

## Author contributions

YA: conceptualization, project administration, literature review, methodology and policy implications, data analysis, methodology, and interpretation of results. GG and JM: project administration, interpretation of results, and supervision. All authors contributed to the article and approved the submitted version.
